# Regulation of Zebrafish Skeletogenesis by *ext2/dackel* and *papst1/pinscher*

**DOI:** 10.1371/journal.pgen.1000136

**Published:** 2008-07-25

**Authors:** Aurélie Clément, Malgorzata Wiweger, Sophia von der Hardt, Melissa A. Rusch, Scott B. Selleck, Chi-Bin Chien, Henry H. Roehl

**Affiliations:** 1 MRC Centre for Developmental and Biomedical Genetics, University of Sheffield, Sheffield, United Kingdom; 2 Department of Biomedical Science, University of Sheffield, Sheffield, United Kingdom; 3 Abteilung Genetik, MPI für Entwicklungsbiologie, Tuebingen, Germany; 4 Department of Pediatrics, University of Minnesota, Minneapolis, Minnesota, United States of America; 5 Department of Genetics, Cell Biology, and Development, University of Minnesota, Minneapolis, Minnesota, United States of America; 6 Department of Neurobiology and Anatomy, University of Utah, Salt Lake City, Utah, United States of America; 7 Brain Institute, University of Utah, Salt Lake City, Utah, United States of America; University of Pennsylvania School of Medicine, United States of America

## Abstract

Mutations in human *Exostosin* genes (*EXT*s) confer a disease called Hereditary Multiple Exostoses (HME) that affects 1 in 50,000 among the general population. Patients with HME have a short stature and develop osteochondromas during childhood. Here we show that two zebrafish mutants, *dackel* (*dak*) and *pinscher* (*pic*), have cartilage defects that strongly resemble those seen in HME patients. We have previously determined that *dak* encodes zebrafish Ext2. Positional cloning of *pic* reveals that it encodes a sulphate transporter required for sulphation of glycans (Papst1). We show that although both *dak* and *pic* are required during cartilage morphogenesis, they are dispensable for chondrocyte and perichondral cell differentiation. They are also required for hypertrophic chondrocyte differentiation and osteoblast differentiation. Transplantation analysis indicates that *dak^−/−^* cells are usually rescued by neighbouring wild-type chondrocytes. In contrast, *pic^−/−^* chondrocytes always act autonomously and can disrupt the morphology of neighbouring wild-type cells. These findings lead to the development of a new model to explain the aetiology of HME.

## Author Summary

Hereditary Multiple Exostoses is a disease that causes the formation of benign bone tumours in children. Besides causing severe skeletal deformity, the bone tumours can compress nerves or other tissue resulting in chronic pain. Although the tumours can usually be surgically removed, they sometimes recur or are in positions that prevent surgery. We have identified two strains of zebrafish whose offspring have skeletal defects that resemble those of patients with Hereditary Multiple Exostoses. We have found that each strain carries a mutated form of an essential gene. Importantly, these two genes are also found in humans, and thus by analysing their function in zebrafish, we may shed light on their role in humans. Our study has elucidated the roles of these genes during normal skeletal development and has allowed us to generate a model for how genetic changes give rise to bone tumours in humans.

## Introduction

Mutations in human *EXT1* and *EXT2* confer an autosomal dominant disorder called HME [1],[2],[3]. Both *EXT1* and *EXT2* encode glycosyltransferases that together form a hetero-oligomeric complex in the Golgi and catalyse the polymerisation of sugars to form heparan sulphate (HS) (for review see [4]). Patients with HME have a short stature and during childhood develop osteochondromas (also called cartilaginous exostoses) that first appear near the growth plate regions of their skeleton. Osteochondromas are made up of a cartilage cap that resembles a growth plate and a bony collar that forms a marrow cavity that is contiguous with the underlying bone. While osteochondromas are normally benign, they can lead to complications and patients have a 1–2% risk of developing chondrosarcoma or osteosarcoma. Most of the tested patients with HME are heterozygous for mutations in either *EXT1* (41%) or *EXT2* (30%) [5]–[7]. Determining the genetic basis for the cases that cannot be attributed to *EXT* genes (29%) is essential for counselling HME patients.

The sporadic and dominant nature of osteochondromas formation in HME patients has led to the proposal of two genetic models (For discussion see [8]). Osteochondromas may arise from a loss-of-heterozygosity (LOH) at one of the *EXT* loci in skeletal cell resulting in unregulated growth and clonal expansion. In support of this model, LOH due to somatic mutations or aneuploidy has been identified in a small number of the osteochondromas analysed [9],[10]. In addition, HS is absent in chondrocytes within osteochondromas which is consistent with a complete loss of EXT function due to LOH [11]. Contrary to this model, HS is secreted and it is likely that a homozygous mutant chondrocyte would be rescued by contact with neighbouring cells. The alternative model is that reduced *EXT* gene dosage causes reduced HS synthesis that results in a structural change in the growth plate. This change allows chondrocytes to occasionally escape normal developmental constraints to give rise to an osteochondroma. The finding that the majority of analysed exostoses do not show a second mutation in the EXT gene family lends support to the gene dosage theory [10]. Resolving between these two models could play an important role in designing future treatment for HME patients.

Skeletal histology in fish is comparable to that of tetrapods [12] and the development of the cranial skeleton of zebrafish has been well described [13],[14]. The precartilage condensations that will give rise to the cartilaginous skeleton begin to appear during the second day of development. Condensations give rise to two cell types: the cells of the perichondrium (a sheath that encapsulates the cartilage) and the chondrocytes that begin to secrete the cartilage matrix. As the skeleton forms, some chondrocytes flatten and intercalate to form a column that gives rise to rod shaped cartilage elements. Alternatively, chondrocytes flatten to form a single layer of tessellated cells that give rise to plate-like elements [15]. Much of the cartilaginous skeleton is then replaced by bone in a process that resembles endochondral ossification in tetrapods. These bones are referred to as cartilage bones. Also, like tetrapods, some of the bony skeleton does not form from a cartilage template. These bones are called intramembranous (or dermal) bones.

Large-scale genetic screens have identified many genes that disrupt skeletal development in zebrafish [16]–[18]. Here we have focused on two genes that are required for skeletal development, *dak* and *pic*. Both *dak* and *pic*, along with a third gene *boxer*, are also required for fin development [19],[20] and axon sorting [21] suggesting that the mutated genes act in a common pathway. We have previously shown that *dak* and *boxer* encode glycosyltransferases required for HS synthesis (*ext2/dak*, *extl3/boxer*) [22]. In this study we present evidence that *pic* encodes a putative PAPS transporter (3′-phosphoadenosine 5′-phosphosulfate transporter, PAPST1) that is required for sulphation of glycans. We show that *dak* and *pic* are required for cartilage morphogenesis, but surprisingly not for early cartilage differentiation. We show that hypertrophic differentiation of chondrocytes and subsequent cartilage bone formation is lost in mutant larvae. We also show that intramembranous bone formation is reduced due to a reduction of osteoblast differentiation. We show that *dak* and *pic* can act cell autonomously during chondrogenesis, and based upon these findings propose a model for how LOH could account for osteochondroma formation in HME patients.

## Results

### Chondrocyte Morphology *dak^−/−^* and *pic^−/−^* Larvae Resembles that Found in HME Patients

**Figure 1:**
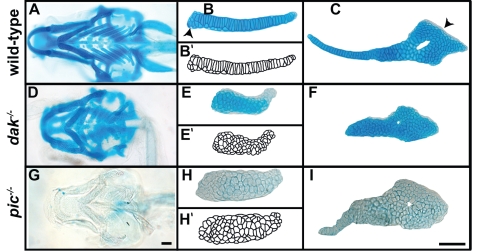
*dak^−/−^* and *pic^−/−^* larvae have similar cartilage morphogenesis phenotypes. Skeletal preparations of wild-type (A–C), *dak^−/−^* (D–F) and *pic^−/−^* (G–I) at day 6 reveal the shape of the cartilaginous skeleton as well as chondrocyte morphology. Ventral views of the head show that the cartilage elements of *dak^−/−^* and *pic^−/−^* fish are shorter and thicker than wild-type (A,D,G). Dissected cartilage laid flat show a complete lack of chondrocyte flattening and intercalation in skeletal elements from *dak^−/−^* and *pic^−/−^* larvae (B,E,H ceratobranchial 1; C,F,I hyosymplectic). Arrowheads in B and C indicate regions that lack stacking in wild-type embryos. Alcian Blue staining at pH1.0 (HCl 0.1N) does not stain *pic^−/−^* cartilage (G,H,I). Camera lucida drawings of chondrocytes in wild-type and mutant larvae (B',E',H'). Scale bars = 50μM.

Chondrocytes in osteochondromas often differ from chondrocytes found in normal growthplates. Instead of being flattened and forming long columns of cells, they are usually rounded and form clusters of cells [11],[23],[24]. We wondered whether chondrocytes in *dak^−/−^* and *pic^−/−^* mutants behave in a similar way. Although most of the cartilage elements are present in both *dak^−/−^* and *pic^−/−^* larvae, the elements are shorter and thicker than wild-type (Figure 1A,D,G) [18]. In *dak^−/−^* larvae, anterior cartilages tend to have more cells than wild-type and posterior cartilages have less. For example, in 144hpf *dak^−/−^* larvae the Meckel's cartilage much larger than in wild-type, while ceratobranchial 4 is small or even absent (H.R. and M.W. unpublished). Wild-type chondrocytes flatten along the longitudinal axis (stack) in most elements (Figure 1A–C) and in rod shaped elements the cells intercalate to form a single column (Figure 1B). Some elements have regions where stacking is not obvious (arrowhead in Figure 1C) especially in regions adjacent to joints (arrowheads in Figure 1B). In all *dak^−/−^* larvae, all chondrocytes are round and do not form into columns (Figure 1D–F). While *pic^−/−^* larvae show lower expressivity, most larvae have a loss of chondrocyte organisation that resembles that seen in *dak^−/−^* larvae (Figure 1G–I). One striking difference between *dak^−/−^* and *pic^−/−^* larvae is that *pic^−/−^* larvae do not stain with Alcian Blue at pH1.0 (Figure 1D,G) but do stain at pH2.5 (M.W. and A.C. unpublished).

### Sulphate Synthesis Is Affected in *pic^−/−^* Embryos

**Figure 2:**
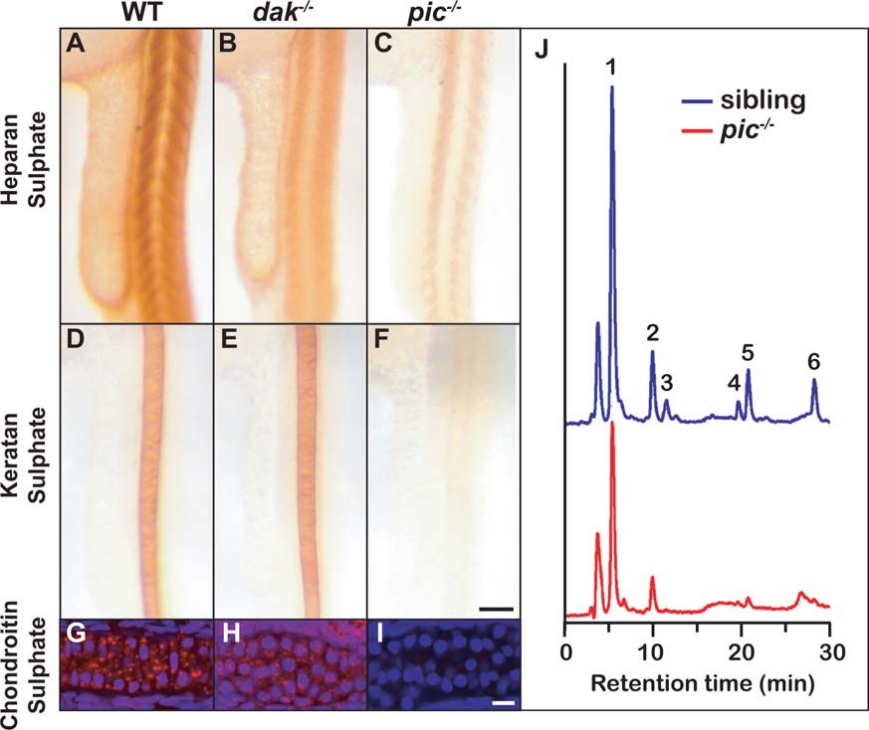
Sulphated proteoglycans are reduced in *dak^−/−^* and *pic^−/−^* larvae. Whole mount antibody staining at 24hpf reveals that HS is reduced in the somites of *dak^−/−^* and *pic^−/−^* (A–C) and KS in the notochord is reduced only in *pic^−/−^* larvae (D–F). Cartilage staining of the ceratohyal at 72hpf reveals that CS is made in wild-type and *dak^−/−^* chondrocytes (red stain in G,H), and absent from *pic^−/−^* cartilage (I) (nuclear staining is shown in blue with DAPI). HPLC analysis of HS in *pic^−/−^* larvae at day 5 indicates that sulphated disaccharides are nearly absent and unsulphated disaccharides are reduced (red) compared to their siblings (blue) (J). 1: ΔUA-GlcNAc, unsulfated Δ^4,5^-unsaturated hexuronate -N-acetyl glucosamine; 2: ΔUA-GlcNS, ΔUA-N-sulfated glucosamine; 3: ΔUA-GlcNAc6S, ΔUA-6-O-sulfated GlcNAc; 4: ΔUA-GlcNS6S, ΔUA-N-sulfated, 6-O-sulfated glucosamine; 5: ΔUA2S-GlcNS, 2-O-sulfated ΔUA-N-sulfated glucosamine; 6: ΔUA2S-GlcNS6S, 2-O-sulfated ΔUA-N-sulfated, 6-O-sulfated glucosamine. These disaccharides correspond to the major disaccharides found in both invertebrate and vertebrate animals. Panel F scale bar = 50μM. Panel I scale bar = 10μM.

As Alcian Blue preferentially stains sulphated groups at low pH [25], one possible explanation for the lack of staining in *pic^−/−^* larvae is a loss of sulphation of glycans and other sulphated moieties. To investigate this further, we used antibodies for HS [26], CS [27] and KS [28] and found that whereas HS is reduced in both *dak^−/−^* and *pic^−/−^* larvae (Figure 2A–C), CS and KS are reduced only in *pic^−/−^* larvae (Figure 2D–I). The antibody used to detect heparin, 10E4, recognizes an epitope that is localised to basal laminae, but not found in the developing zebrafish cartilage (Figure S2). We further analysed HS composition using heparan lyase digestion followed by HPLC [22]. Peaks generated by zebrafish larval extracts were compared to 6 known standards. *pic^−/−^* embryos show a severe reduction of sulphated disaccharides, but surprisingly also show a reduction in unsulphated disaccharides (Figure 2J). This perhaps indicates that the loss of sulphation affects processing or stability of heparan. However, it is important to note that in the absence of sulphation, heparan synthesis may generate atypical disaccharides which would not be identified by this analysis [29]. Together these data confirm that *pic* is required for sulphation of proteoglycans.

### *pic* Encodes a PAPST1, a New Candidate Gene for HME

**Figure 3:**
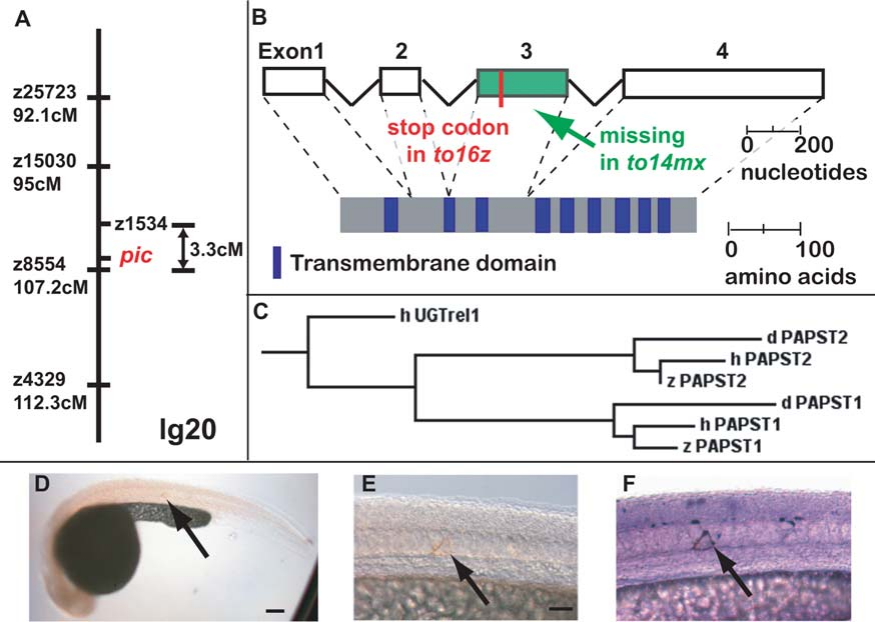
*pic^−/−^* encodes a PAPS transporter. Genetic map of *pic* locus (A). Note that z1534 has not been assigned a genetic position as it was found on the RH map. Allele sequencing identifies *pic^to216z^* as a premature stop codon in exon 3 and *pic^to14mx^* as a deletion of exon 3 (B). Zebrafish PAPST1 clusters with human and Drosophila PAPST1 in a dendrogram (C). Expression of wild-type PAPST1 in a single notochord cell rescues synthesis of KS in *pic^−/−^* larvae (D–E). Close-up of single cell secreting KS in brown (arrow in E), and the same cell counterstained for the rescue construct reporter in purple (arrow in F). The dendrogram was made using Clustal W and Treeview68k. Accession numbers: AB107958, NP_648954, AB106538, NP_057032, NP_991198, NP_001035084, NM_005827. Zebrafish and human PAPST1 share 70% amino acid identity. Panel D scale bar = 100μM. Panel E scale bar = 50μM.

29% of patients with HME do not carry mutations in EXT1 or EXT2 genes. In order to help identify new candidate genes, we positionally cloned *pic*. Using SSLP microsatellite markers, we mapped the *pic* locus to a 3.3cM interval on chromosome 20 (Figure 3A). Using the zebrafish RH map, we placed a zebrafish gene with homology to human and Drosophila *PAPST1* [30],[31] in the same interval (Figure 3C and Table S1). As PAPST1 transports PAPS into the Golgi (PAPS being the universal donor for sulphation), it is a good candidate gene to explain the loss of proteoglycan sulphation. We then sequenced *papst1* cDNA from *pic^to216z/to216z^* and *pic^to14mx/to14mx^* mutant embryos (Figure 3B). The *pic^to216z^* allele has a nucleotide transition (G to A) at position 390 in the third exon, creating a stop codon. The *pic^to14mx^* allele is a genomic deletion that results in an in-frame deletion of all of exon 3 in the cDNA. To confirm that mutations in *papst1* result in the *pic^−/−^* phenotype, we expressed the wild-type zebrafish *papst1* cDNA under the control of a heterologous promoter in *pic^−/−^* embryos. Expression of wild-type *papst1* in a single cell was sufficient to rescue staining of KS in the notochord (Figure 3D–F). To determine the expression pattern of *papst1*, we performed wholemount in situ hybridization with the full-length cDNA. Consistent with its role as a general component of the cellular sulphation machinery, *papst1* is expressed ubiquitously (Figure S1). As both alleles are predicted to result in severe truncation of the PAPST1 protein and have identical phenotypes, they are likely to be null alleles.

### The Onset of Cartilage Differentiation Is Normal in *dak^−/−^* and *pic^−/−^* Larvae

**Figure 4:**
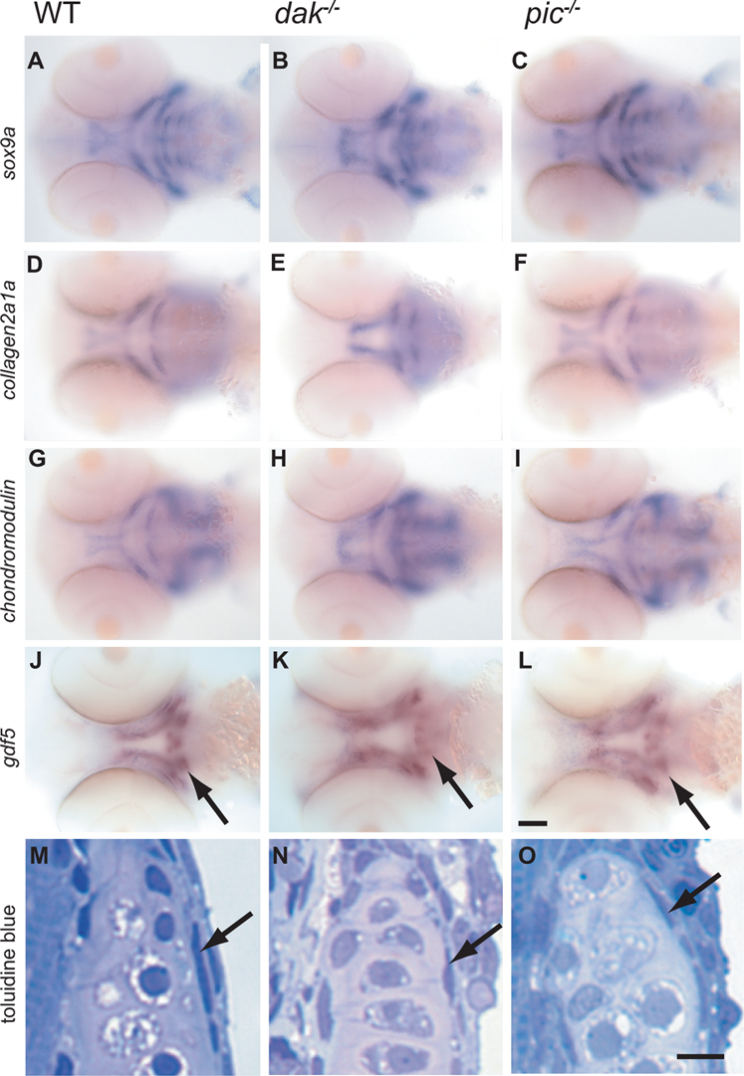
*dak^−/−^* and *pic^−/−^* larvae have wild-type levels of expression of markers of chondrocyte differentiation. Wholemount RNA in situ analysis of *sox9a* (A–C), *collagen2a1a* (D–F), *chondromodulin* (G–I) and *gdf5* (J–L) at 60hpf (all ventral views of the head). Although the position of the developing skeleton varies between wild-type and mutants, the markers are expressed at similar levels in wild-type (A,D,G,J), *dak^−/−^* (B,E,H,K) and *pic^−/−^* (C,F,I,L). *dak^−/−^* larvae express *sox9a* at higher levels anteriorly, but this is perhaps due to more chondrogenic cells being present (see Figure 6). Expression of *gdf5* in the perichondrium of the ceratohyal is present albeit slightly reduced in *dak^−/−^* and *pic^−/−^* (arrows in J–L). The perichondrium of the hyosymplectic is also seen in toluidine blue stained sections at day 5 (arrows in M,N,O). Panel I scale bar = 50μM. Panel L scale bar = 5μM.

The LOH model for HME raises the question of whether *EXT^−/−^* cells could differentiate into all the cell types that make up an osteochondroma. To address this question, we first tested whether perichondral cells and chondrocytes differentiate normally in *dak^−/−^* and *pic^−/−^* mutant larvae. Surprisingly, expression of three markers of early chondrogenesis occurs in both mutants as in wild-type larvae (Figure 4A–I). To test whether the perichondrium is present, we used a marker, *gdf5*, which is expressed in the perichondrium of the ceratohyal [32]. Expression is present in the ceratohyal of both mutants (arrows in Figure 4J–L). The flattened cells of the perichondrium can also be seen in toluidine blue stained sections of mutant larvae, indicating that HS is dispensable for the differentiation and morphogenesis of these cells (arrows in Figure 4M–O). Together, these data suggest that the cartilage and perichondral components of osteochondromas could be formed by *EXT^−/−^* cells.

### Ossification Is Reduced in both *dak^−/−^* and *pic^−/−^* Larvae

**Figure 5:**
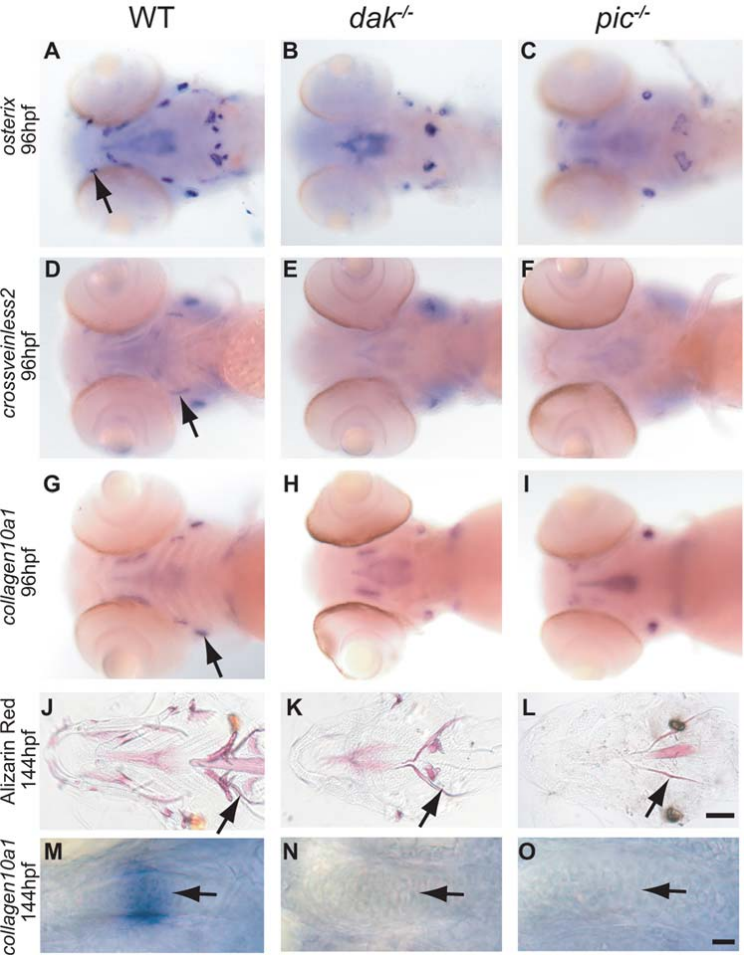
Reduction of bone development in *dak^−/−^* and *pic^−/−^* larvae. Wholemount RNA in situ analysis of *osterix* (A–C), *crossveinless* (D–F) and *collagen10a1* (G–I) at 96hpf (all ventral views of the head). Markers for dermal and cartilage bone development are down-regulated or absent in both mutants (A,D,G wild-type; B,E,H *dak^−/−^*; C,F,I *pic^−/−^*). Arrows indicate wild-type expression in the maxilla (A), branchiostegal ray (D) and opercle (G). The loss of marker gene expression is consistent with the later reduction in bone formation in ventral views of 6 day old larvae stained with Alizarin Red (J,K,L). Arrows indicates the location of the cleithrum in J, K and L. Table S2 lists all of the affected bones. *collagen10a1* expression in chondrocytes of the ceratohyal in wild-type larvae marks chondrocytes as they become hypertrophic (arrow in M). This expression is absent in *dak^−/−^* (N) and *pic^−/−^* (O) larvae. Panel L scale bar = 100μM. Panel O scale bar = 10μM.

As osteochondromas contain a bony collar, we next tested whether bone forms normally in homozygous *dak^−/−^* and *pic^−/−^* larvae. Intramembranous (dermal) and cartilage bones appear during early larval development [13]. Alizarin Red staining for bone at 144hpf shows a strong reduction of calcification in both bone types (Figure 5J–L). Consistent with this, there is a strong reduction of several markers for osteoblast differentiation in both mutants at 96hpf (Figure 5A–I and see Table S2). As cartilage hypertrophy precedes endochondral ossification, we also tested whether expression of the hypertrophic marker, *collagen10a1* is affected at 144hpf. We found that chondrocyte expression of *collagen10a1* is absent in both mutants (arrows in Figure 5 M–O). Together these data suggest that *EXT^−/−^* cells in an osteochondroma would not contribute significantly to the formation or remodelling of bone. Thus it is likely that *EXT^−/+^* cells would be recruited to an osteochondroma and take part in the formation of the bony collar.

### Pre-Cartilage Condensations form but Chondrocyte Cell Behaviour Is Deficient in *dak^−/−^* Embryos

**Figure 6:**
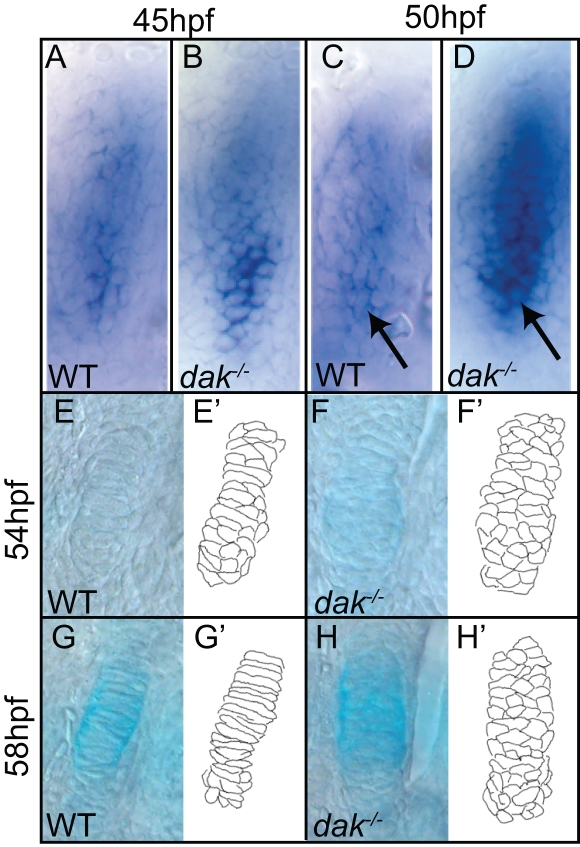
Condensation morphogenesis is absent in *dak^−/−^* larvae. Pre-cartilage condensations in the first arch are larger in *dak^−/−^* larvae as judged by *sox9a* staining at 45hpf (A,B) and 50hpf (C,D). Individual cells within the condensation appear to have a more rounded morphology in *dak^−/−^* larvae than in wild-type larvae (compare arrows in C,D). Intercalation and flattening of chondrocytes to give rise to the ceratohyal cartilage in the second arch takes place between 54 and 58hpf in wild-type (E,G), but does not occur in *dak^−/−^* larvae (F,H). Chondrocytes begin to secrete cartilage matrix during this time as seen by Alcian Blue staining (G,H). Camera lucida drawings of chondrocytes in wild-type and *dak^−/−^* larvae (E'–H'). Scale bars = 10μM.

To determine when chondrocyte behaviour is first affected in *dak^−/−^* embryos, we looked at condensation formation in the jaw. Early condensations within the first arch were visualized at 45 and 50hpf using *sox9a* as a chondrogenic marker (Figure 6A–D). Even at this early stage, *dak^−/−^* chondrocytes appeared more round than those in wild-type embryos (as judged by nuclear morphology, arrows in Figure 6C,D). In anterior condensations, the level of *sox9a* expression is variable and usually stronger in *dak^−/−^* condensations, consistent with the increase in chondrocyte cell number seen in anterior arches (Figure 6B,D). We also examined early stacking within condensations of the second arch at 54 and 58hpf. During this time wild-type chondrocytes intercalated to form a single cell layer, flattened perpendicular to the growth axis and began to secrete cartilage matrix (Figure 6E,E',G,G'). Although *dak^−/−^* chondrocytes also began secreting matrix, the cells showed no signs of undergoing morphogenesis (Figure 6F,F',H,H'). Taken together, these data suggest that the primary cartilage defect in *dak^−/−^* larvae is the loss of chondrocyte organisation. Similar results were obtained with *pic^−/−^* larvae but with lower and more variable expressivity (data not shown).

### *dak^−/−^* Chondrocytes Show both Autonomous and Non-Autonomous Behaviour

**Figure 7:**
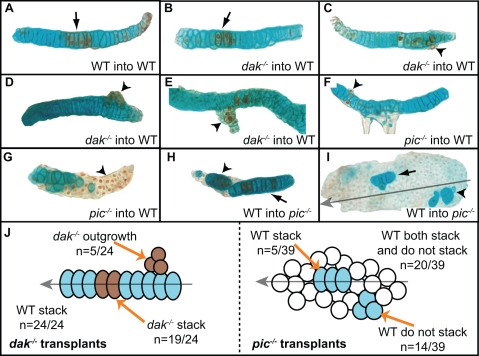
Cell autonomous behaviour of *dak^−/−^* and *pic^−/−^* chondrocytes. Transplanted *dak^−/−^* cells usually form columns with wild-type chondrocytes (arrow in B). However, in some cases *dak^−/−^* cells grow out from the wild-type host cells and behave autonomously (arrowheads in C,D,E). *pic^−/−^* cells transplanted into wild-type hosts never stack (arrowheads in F,G). In addition, wild-type cells both stack (arrows in H,I) and fail to stack (arrowheads in H,I) when transplanted into *pic^−/−^* hosts. Wild-type cells that stack in *pic^−/−^* hosts form columns that lie parallel to the longitudinal axis (grey arrow in I). (A–I) Dissected cartilage elements; all are ceratobrachial cartilage except (E) and (I) which are trabecular and ceratohyal cartilage respectively. Brown cells in (A–G) are transplanted cells, blue cells in (H,I) are transplanted cells. (J) summarises all 63 transplants analysed. Transplanted cells that flattened also intercalated to form columns. WT = wild-type.

One caveat in the LOH model is that HS is secreted and thus an *EXT^−/−^* cell that arises would be rescued by neighbouring cells. To ascertain whether clones of *dak* ^−/−^ cells behave autonomously when juxtaposed to HS secreting cells, we transplanted *dak^−/−^* cells into wild-type embryos. The transplantations were done at sphere stage, then the embryos were allowed to develop for several days before fixation and analysis. We found that in most cases (19/24 transplants), the transplanted mutant cells stacked normally when juxtaposed to wild-type cells (arrow, Figure 7B). This alone would argue that single *EXT^−/−^*cells in HME patients would be unable to form exostoses and thus would refute the LOH model. However in some cases (5/24) mutant cells behaved autonomously and failed to stack or intercalate (arrowheads, Figure 7C–E). These mutant clones grew out from the edge of the cartilage perpendicular to the wild-type stacks. Given that HS may not diffuse far, it is plausible that *dak^−/−^* cells on the edge of the cartilage lack sufficient contact to be rescued by neighbouring wild-type chondrocytes. This explanation is consistent with studies of HME patients that have found that osteochondromas are first seen on the edge of the growth plate just beneath the perichondrium [33],[34]. Importantly, this results shows that cells that *ext2^−/−^* chondrocytes can behave autonomously in zebrafish and thus *EXT^−/−^* chondrocytes in humans could be responsible for the formation of osteochondromas.

### *pic^−/−^* Chondrocytes Have Autonomous Behaviour

In all transplants examined (39/39), *pic^−/−^* cells behaved autonomously and failed to stack or intercalate (arrowheads, Figure 7F,G). In addition, when juxtaposed to *pic^−/−^* cells, wild-type cells often adopted the mutant rounded morphology (arrowheads, Figure 7H,I). In many cartilage elements, both stacked and non-stacked clusters of wild-type chondrocytes were seen (20/39). Significantly, whenever wild-type cells stacked, they flattened and formed columns that were oriented to the longitudinal axis of the cartilage element, even when few wild-type cells were present in a *pic^−/−^* cartilage element (arrow, Figure 7I). This suggests that there is a signal that polarizes chondrocytes so that stacking is oriented to the correct axis and that this signal is still present in *pic^−/−^* larvae. These findings are also consistent with the LOH model and suggest that patients with mutations in PAPST1 may have more severe clinical symptoms.

## Discussion

### The LOH Model for Osteochondroma Formation

**Figure 8:**
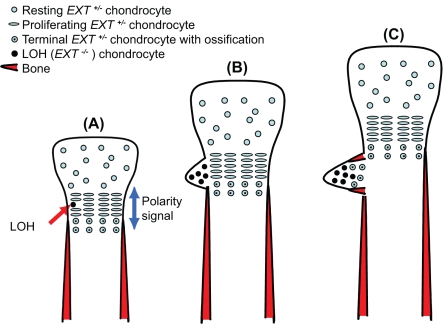
LOH model for osteochondroma formation. (A) *EXT^−/−^* chondrocytes that originate or end up on the edge of the growth plate lose the ability to respond to a polarity signal. These chondrocytes adopt a round morphology and do not contribute to the neighbouring columns of chondrocytes. The LOH may occur during early skeletal development, before formation of the bony collar. (B) As *EXT^−/−^* chondrocytes proliferate they form a clone of cells that begin to grow out from skeleton beneath the *EXT^−/+^* perichondrium. (C) *EXT^−/+^* chondrocytes join the clone of *EXT^−/−^* cells, express *collagen10* and become hypertrophic. *EXT^−/+^*osteoblasts are recruited to form the bony collar. This results in the cartilage cap being predominantly composed of *EXT^−/−^* chondrocytes, while the cells that make up the rest of the osteochondroma (hypertrophic chondrocytes, osteoblasts and perichondrial cells) are predominantly *EXT^−/+^*.

While much is known about the genetic basis of HME, the mechanism of osteochondroma formation is poorly understood. In this study, we show that zebrafish is an excellent model for HME and our findings support the LOH model in several ways. First, proliferating chondrocytes in osteochondromas resemble those seen in homozygous *dak^−/−^* larvae: they are rounded and do not form into columns of cells [11],[23],[24]. Second, we show that homozygous mutant cells differentiate into chondrocytes, despite the absence of morphogenesis. Third, transplants with *dak^−/−^* cells into wildtype animals show that although most homozygous mutant clones were rescued, some *dak^−/−^* chondrocytes behaved autonomously. The rescue of mutant cells is presumably due to HS secretion from neighbouring wild-type cells, but may also be due to other secreted factors. The results presented here as well as from other studies suggest a model for how LOH could result in osteochondroma formation (Figure 8). Although our results lend credence to the LOH model, they do not refute the gene dosage model and it is possible that both mechanisms play a role.

### Different Phenotypes in Mouse and Zebrafish Models for HME

Several studies of the role of EXT genes during mouse skeletogenesis have been published and these favour the gene dosage model for HME. In mice homozygous for a hypomorphic allele of *Ext1* (*Ext1^gt/g^* ^t^) or heterozygous for a targeted deletion (*Ext1^+/−^*), the chondrocytes of limb growth plates show delayed hypertrophic differentiation and endochondral ossification [35],[36]. Given that HS is known to regulate the activity of many signalling pathways, the researchers tested whether a signalling defect could explain the *Ext1* mutant phenotype. Indian Hedgehog (IHH), a signalling protein that normally acts in the growth plate to block hypertrophy and terminal differentiation of chondrocytes was found to have increased activity in mutant mice. The model for these results is that in wild-type animals, HS normally acts to limit diffusion of IHH and thereby allow chondrocytes to become hypertrophic [35],[36]. The authors favour the gene dosage model for HME and propose that hereditary osteochondromas are caused in part by a delay in chondrocyte hypertrophy caused by excessive IHH signalling [35],[36]. In contrast, mice heterozygous for a targeted deletion of *Ext2* (*Ext2^+/−^*) have normal limb growth plates and there is no discernible effect on IHH diffusion [37]. However, *Ext2^+/−^* mice do have osteochondroma-like outgrowths on their ribs. These authors also favour the gene dosage model but do not find evidence to support a role for IHH.

In comparison, *dak^−/−^* and *pic^−/−^* larvae show a more severe skeletal phenotype, perhaps due to a stronger reduction of HS. Whereas mice homozygous for null mutations in *Ext1* or *Ext2* in mice arrest during gastrulation [37],[38], *dak^−/−^* and *pic^−/−^* embryos can gastrulate probably due to maternally deposited RNAs ([22] and Figure S1). By 36hpf, HS is only weakly detectable in *dak^−/−^* and *pic^−/−^* embryos (by immunohistochemistry, AC and MW unp). The early reduction of HS has enabled us to identify cartilage morphogenesis as the primary defect during skeletogenesis. Indeed, chondrocytes in both *Ext1^gt/g^* ^t^ and *Ext2^+/−^* mice show a mild disruption of the columnar organization within the growth plate [36],[37]. Thus it is likely that complete loss of stacking, as early as the cartilage condensation phase, would be evident with a more severe reduction of mouse *Ext* gene function.

IHH signalling is not likely to be responsible for the cartilage morphogenesis phenotype because neither of the two zebrafish IHH genes is expressed until 2 days after chondrocyte stacking begins. Furthermore, pharmacological inhibition of Hedgehog signalling during skeletogenesis does not affect chondrocyte stacking (MW and AC unpublished). One plausible candidate for this signal in zebrafish is *wnt5b* which encodes a ligand for the non-canonical WNT signalling pathway [39]. The evidence for this is that *wnt5b* is expressed in cells surrounding cartilage condensations and mutations in *wnt5b* result in reduced chondrocyte stacking (MW and AC unpublished). As the *wnt5b* ^−/−^ cartilage phenotype is mild compared to that of *dak^−/−^* larvae, other members of the non-canonical WNT family of genes may be redundant with *wnt5b*. An intriguing possibility is that WNT signalling and other components of the planar cell polarity system mediate chondrocyte stacking, just as they regulate convergence/extension movements during gastrulation.

Although early chondrocyte differentiation is unaffected in zebrafish mutant larvae, we did find that expression of the hypertrophic marker, *collagen10a1*, is lost. This is in agreement with results from *Ext1* mutant mice which show a delay in chondrocyte hypertrophy due to increased IHH signalling [35],[36]. The opposite result has been found for *Ext2* mutant mice where a reduction in HS was shown to cause premature *collagen10* expression [37]. Intriguingly, studies of HME patients have found evidence of premature hypertrophy in osteochondromas [23],[24]. Determining why apparently conflicting results have been obtained in these systems will require more detailed analysis.

HS has been implicated in osteoblastogenesis, however there has been no clear evidence for a developmental role to date [40]. Here we show that osteoblastogenesis is impaired by the reduction of HS mutant zebrafish larvae. Although previous *Ext* mutant mouse studies have focused on cartilage differentiation, a reduction in the bone mineral density of *Ext1^+/−^* mice has been observed [35]. Furthermore, osteopenia has been shown to be associated with HME in a family that carries a mutation in *EXT1* [41]. These findings suggest a new role for HS during osteoblast differentiation.

### PAPST1 as a Candidate Gene for HME

While most of the tested patients with HME are heterozygous for mutations in either *EXT1*(41%) or *EXT2* (30%), the genetic basis of the remaining cases is unknown (29%) [5]–[7]. Several *EXT-like* genes have been shown encode enzymes required during HS synthesis and would thus make good candidate genes (*EXTL1*, *EXTL2*, and *EXTL3*). Unfortunately, none of these have been shown to carry disease-related mutations in HME patients [7]. Although mutations in sulphate metabolism can cause hereditary skeletal disorders, none of these results in the formation of osteochondromas [42]–[44]. Here we show for the first time that *PAPST1* is essential for sulphation of glycans in vertebrates and that mutations in *pic* confer a specific phenotype that is very similar to the *dak^−/−^* phenotype. This is a surprising result because unlike *dak* mutations which only reduce HS, *pic* mutations should reduce all sulphation in the cell. Inactivation of *PAPST* genes in Drosophila also results in phenotypes that resemble *EXT* mutant phenotypes [31],[45]. Together, these findings suggest that although other glycans may be required during vertebrate and invertebrate development, HS is the principal glycan. Furthermore, our results suggest that *PAPST1* as well as other genes involved with sulphate transport and metabolism are candidate genes for HME.

## Materials and Methods

Unless otherwise stated, all methods were based upon standardised protocols [46]. For *dak* mutant analysis, allele *to273b* was used for all analysis, as it should result in a complete loss of Ext2 function [22]. *dak^−/−^* larvae were identified by the absence of fins. For *pic* analysis, allele *to14mx* was used unless otherwise stated. To identify *pic* ^−/−^ larvae before the phenotype is morphologically visible, genomic DNA was extracted, and PCR was performed using TAQ polymerase and the following primers: F2: 5′CGT GTG ATG ACG CGC TCA TAC 3′

R1ab: 5′AGC GCC AGG ATG CGG TTC AT 3. The conditions were 94°C (30 seconds), 55°C (60 seconds) and 72°C (60 seconds) for 35 cycles. DNA from homozygous mutants does not generate a band as the 14mx mutation deletes this region.

### Genetic Mapping and Cloning of *pic*

The *pic* locus was mapped to linkage group 20 (lg20) after analysing SSLP (simple sequence length polymorphism) markers on 700 meioses. *pic^to14mx^* maps 2.7cM south of z1534 (20/740 meiosis) and 0.6cM north of z8554 (4/698 meiosis) [47]. This interval on the T51 radiation hybrid (RH) map [48] was found to contain many ESTs. A contig in the neighborhood of one of these, fc88f04.x1, was assembled using traces from the Sanger Centre, and found to contain a gene having homology to a human and Drosophila *PAPS Transporter 1* (*PAPST1*, also known as: *Solute Carrier Family 35, Member B2*) [30],[31]. To confirm the location of zebrafish *papst1*, primers were designed to exon 4 and analysed using the T51 panel (papstf3, 5′ CGTCACCACATTCTCCGGCGT 3′ and papstr3, 5′GTGTCTGATTTCCTGAAGTGT 3′). The *papst1* pattern matches the pattern of other markers in the *pic* interval (see Table S1). To sequence alleles, cDNA was obtained from *pic^to14mx/to14mx^*, *pic^to216z/to216z^* larvae and wild-type larvae and then sequenced with primers papstf1 and papstf1.1e (respectively, 5′ TGGCAGTTTTGTAGAGGCGGAG 3′ and 5′ GAATGCAGACGCTGTAGAC 3′).

### Wholemount Antibody and mRNA *in situ* Staining

Primary antibodies were anti-HS 1∶500 (10E4, Europa), anti-keratan sulphate 1∶100 (KS) (3H1, Developmental Studies Hybridoma Bank), anti-chondroitin sulphate 1∶100 (CS) (CS-56, Sigma), anti-collagen type II 1∶200 (II-II6B3, Developmental Studies Hybridoma Bank) and anti-GFP 1∶100 (Torrey Pines Biolabs). Secondary antibodies were horse anti-mouse-HRP and goat anti-rabbit-HRP (Vector Laboratories). Detection was done using DAB substrate (Vector Laboratories) or TSA-CY3 substrate (Perkin Elmer). Larvae were mounted in 70% glycerol or Vectashield with DAPI (Vector). Antisense probes were made using the following cDNAs: *chondromodulin1* [49], *collagen2a1a* [50], *collagen10a1* [51], *growth and differentiation factor-5* (*gdf5/contact*) [32], *crossveinless2* [52] and *sox9a* [53]. Cloning and characterisation of zebrafish *osterix* will be described elsewhere. Goat anti-DIG fab fragments and NBT/BCIP substrate (Roche) were used to develop the *in situs.*

### Rescue of Keratan Sulphate Synthesis in *pic^−/−^ embryos*

To rescue *pic^−/−^* embryos, we made an expression construct by cloning the wild-type zebrafish *papst1* cDNA into an hspIG vector that contains a heat shock promoter and an IRES2 (internal ribosome entry site)/eGFP cassette (gift from Dr Florian Maderspacher). The construct was then injected into *pic^−/−^* larvae and the wild-type *papst1* cDNA was expressed by heat shocking larvae at 24 hours post-fertilisation (hpf), for 1 hour at 38°C. The larvae were fixed 6 hours later and a double antibody staining was performed to check for rescue. As DNA injected at the one cell stage is inherited mosaically, the eGFP reporter was used to confirm that cells that synthesise keratan sulphate (KS) indeed carried the rescuing construct. First the larvae were stained using anti-KS with DAB as the substrate. After photographing, the embryos were then stained using anti-GFP with NBT/BCIP as the substrate to determine which cells carried the construct.

### Cell Transplantation

GFP donor embryos were first injected with tetramethyl-rhodamine dextran 3% (Invitrogen) at 1-cell stage. Then, both donors and recipients were dechorionated in pronase. Transplantation was done in E3 from sphere stage and based upon a zebrafish fate map (Woo and Fraser, 1995). At 24hpf, each recipient was screened for the presence of fluorescent rhodamine in the neural crest cells and kept until 120hpf with their donor. Larvae were then fixed in 4% PFA and Alcian Blue staining followed by antibody staining to track the GFP transplanted cells was performed. The experiment was done with *pic^to216z/to216z^* or *dak^to273b^* ^/*to273b*^ transplanted into wild-type and vice versa.

## Supporting Information

Figure S1 *papst1* is expressed ubiquitously. mRNA *in situ* analysis of *papst1* expression: antisense (A–C) and sense (A prime–C prime) probes at the 30–60 cells stage (A, A prime), 50% epiboly stage (B, B prime) and 7 somites stage (C, C prime).
(1.51 MB EPS)

Figure S2 The 10E4 epitope is not expressed in the developing cartilage. Wildtype (A,C,E) and *pic^-/-^* (B,D,F) localisation of the 10E4 epitope (in green) at 60hpf. Cartilage (collagen type II) is shown in red and nuclei in blue (C–F). Side views of the whole fish (A,B), ventral views of the head (C,D) and high magnification pictures of the ceratohyal (E,F). The 10E4 epitope is detected predominantly on basal laminae but is undetectable in the developing cartilage at the time of chondrocyte stacking. Panel D scale bar = 100μM. Panel F scale bar = 10μM.
(9.71 MB EPS)

Table S1 *papst1* is physically linked to the *pinscher* genetic interval. PCR analysis of *papst1* on the 94 hybrid cell lines from the T51 panel indicates that the majority of positive cell lines are also positive with SSLP markers that are genetically linked to *pinscher.*
(0.93 MB EPS)

Table S2 Expression of bone markers as well as Alizarin Red staining is reduced in *dak^-/-^* and *pic^-/-^* larvae. Scoring was based upon larvae shown in Figure 5. An X indicates that expression is detectable, however expression was often reduced compared to wildtype.
(0.71 MB EPS)
